# A Machine Learning Model to Predict Patients’ Adherence Behavior and a Decision Support System for Patients With Metastatic Breast Cancer: Protocol for a Randomized Controlled Trial

**DOI:** 10.2196/48852

**Published:** 2023-12-14

**Authors:** Marianna Masiero, Gea Elena Spada, Virginia Sanchini, Elisabetta Munzone, Ricardo Pietrobon, Lucas Teixeira, Mirtha Valencia, Aline Machiavelli, Elisa Fragale, Massimo Pezzolato, Gabriella Pravettoni

**Affiliations:** 1 Department of Oncology and Hemato-oncology University of Milan Milan Italy; 2 Applied Research Division for Cognitive and Psychological Science European Institute of Oncology IRCCS Milan Italy; 3 Division of Medical Senology European Institute of Oncology IRCCS Milan Italy; 4 SporeData Inc Durham, NC United States

**Keywords:** adherence, metastatic breast cancer, decision-making, personality, risk-predictive model, decision support system, oral therapies, machine learning, behavior, cancer, breast cancer

## Abstract

**Background:**

Adherence to oral anticancer treatments is critical in the disease trajectory of patients with breast cancer. Given the impact of nonadherence on clinical outcomes and the associated economic burden for the health care system, finding ways to increase treatment adherence is particularly relevant.

**Objective:**

The primary end point is to evaluate the effectiveness of a decision support system (DSS) and a machine learning web application in promoting adherence to oral anticancer treatments among patients with metastatic breast cancer. The secondary end point is to collect a set of new physical, psychological, social, behavioral, and quality of life predictive variables that could be used to refine the preliminary version of the machine learning model to predict patients’ adherence behavior.

**Methods:**

This prospective, randomized controlled study is nested in a large-scale international project named “Enhancing therapy adherence among metastatic breast cancer patients” (Pfizer 65080791), aimed to develop a predictive model of nonadherence and associated DSS and guidelines to foster patients’ engagement and therapy adherence. A web-based DSS named TREAT (treatment adherence support) was developed using a patient-driven approach, with 4 sections, that is, Section A: Metastatic Breast Cancer; Section B: Adherence to Cancer Therapies; Section C: Promoting Adherence; and Section D: My Adherence Diary. Moreover, a machine learning–based web application was developed to predict patients' risk factors of adherence to anticancer treatment, specifically pertaining to physical status and comorbid conditions, as well as short and long-term side effects. Overall, 100 patients consecutively admitted at the European Institute of Oncology (IEO) at the Division of Medical Senology will be enrolled; 50 patients with metastatic breast cancer will be exposed to the DSS and machine learning web application for 3 months (experimental group), and 50 patients will not be exposed to the intervention (control group). Each participant will fill a weekly medication diary and a set of standardized self-reports evaluating psychological and quality of life variables (Adherence Attitude Inventory, Beck Depression Inventory-II, Brief Pain Inventory, 13-item Sense of Coherence scale, Brief Italian version of Cancer Behavior Inventory, European Organization for Research and Treatment of Cancer Quality of Life 23-item Breast Cancer-specific Questionnaire, European Organization for Research and Treatment of Cancer Quality of Life Questionnaire, 8-item Morisky Medication Adherence Scale, State-Trait Anxiety Inventory forms I and II, Big Five Inventory, and visual analogue scales evaluating risk perception). The 3 assessment time points are T0 (baseline), T1 (1 month), T2 (2 months), and T3 (3 months). This study was approved by the IEO ethics committee (R1786/22-IEO 1907).

**Results:**

The recruitment process started in May 2023 and is expected to conclude on December 2023.

**Conclusions:**

The contribution of machine learning techniques through risk-predictive models integrated into DSS will enable medication adherence by patients with cancer.

**Trial Registration:**

ClinicalTrials.gov NCT06161181; https://clinicaltrials.gov/study/NCT06161181

**International Registered Report Identifier (IRRID):**

DERR1-10.2196/48852

## Introduction

### Adherence to Oral Anticancer Treatments

Metastatic breast cancer (MBC) is an incurable disease, wherein the available medications are primarily focused on deferring disease progression and symptom mitigation, thereby prolonging survival rate as well as preserving the quality of life (QoL) and psychological well-being [1,2]. The clinical advancements achieved in anticancer treatments have increased the survival rates of patients with MBC. The 5-year survival rate of patients with MBC is around 38% [2]. Notwithstanding, several studies [3-6] have shown that adherence to anticancer treatments is a critical issue in the disease trajectory of patients with breast cancer, especially regarding oral anticancer treatments (OATs), which are intensely demanding because patients are responsible for assuming medications according to the medical prescriptions, thereby increasing the risk of not appropriately taking the therapy [7].

From a theoretical perspective and according to the World Health Organization’s recommendations [8], medication adherence might be explained by a set of mutual and interconnected determinants incorporating (1) sociodemographic (eg, age, gender, socioeconomic status), psychocognitive, and social variables (eg, psychological well-being, social support); (2) disease and treatment-related characteristics (eg, cancer stage, prognosis, dosage, side effects); (3) attitudes, beliefs, and values; (4) health literacy and knowledge; and finally (5) health care system–related factors [7,9,10]. Accruing evidence have highlighted that patients with advanced cancer might report significant levels of nonadherence because in case of some cancers such as MBC, the patients have to change the type of treatments or dosage frequently and they experience a high level of fear of cancer spread [3]. For example, Yerrapragada and colleagues [6] reported nonadherence to tamoxifen in patients with MBC, ranging between 30% and 85% and further reducing over time. In addition, patients with MBC reported several barriers to the daily management of OAT, such as emotional (eg, worry, depression) and physical distress related to the side effects (eg, fatigue, weakness, sleep disturbance, emotional burden, pain) and lack of knowledge about their disease. Further, patients might experience a lack of control and a lack of perceived benefits during the disease pathway, and they may experience difficulty in managing therapy [3,6]. Other studies have shown that patients with MBC experience modest QoL due to the related treatment side effects, financial burden affecting therapy discontinuation, and a significant level of nonadherence [4,7]. Marshall and colleagues [4] observed that patients with MBC who frequently report treatment concerns rather than treatment benefits are less likely to be adherent to prescriptions, as well as patients who experience a more significant number and severity of side effects tend to have more medication worries that could negatively impact adherence.

### Risk-Predictive Models and Decision Support Systems

Given the impact of nonadherence on clinical outcomes and the associated economic burden on the health care system [11,12], finding effective ways to increase treatment adherence is particularly relevant. Patients who adopt nonadherent behaviors need support in managing oral therapies and in overcoming individual and systemic barriers and roadblocks [5]. Notwithstanding, the dynamics influencing adherence to OATs are understudied in the cancer field [13], and a comprehensive model of the risk determinants of nonadherence is not currently available, particularly for targeted therapies and novel generations of hormonal therapy [14]. Besides, a shared definition of medication adherence and satisfactory assessment tools is not acknowledged, which vary in terms of accuracy and reliability [14-17], even in the explicit case of the equivalent treatment protocol and diagnosis [13,18]. Because of the direct costs of nonadherence (eg, survival rate, health-related QoL for patients with MBC) and the indirect costs for the health care system (eg, economic burden), it is mandatory to identify potential risk factors of nonadherence to OATs and to define personalized interventions supporting adherence during the clinical pathway in patients with MBC.

Consistently, defining, measuring, and developing a comprehensive model of medication adherence based on real-world data predictive models is a crucial clinical, psychological, social, and economic challenge. Machine learning has become an integral part of the health care industry—from biomedical research to the delivery of health care services. Compared to traditional statistical methods, machine learning provides numerous advantages such as increased flexibility, prediction accuracy, possibility of automation, and processing of big data. Prediction models for adherence have already been developed and tested in various scenarios [19-22]. If adequately reported, these models can help guide treatment decision-making, improve patient outcomes, and streamline perioperative health care management. Considering the complexity of medication nonadherence in patients with MBC, it is critical to identify patients at risk of nonadherence and carry out timely, precise, and tailored interventions to improve their adherence. Through machine learning models, it is possible to provide personalized prediction on medication adherence for a given patient, supporting adherence and performing a specific intervention [22,23].

A growing body of studies have underlined that eHealth technologies (eg, mobile apps, web-based solutions, wearables) might be helpful tools to foster patient management and engagement in clinical decisions during the cancer pathway [24]. Different web-based solutions based on educational and behavioral interventions have been developed for patients with breast cancer to foster medication adherence [25,26]. For example, the Multinational Association for Supportive Care in Cancer has developed an educational and teaching tool for patients with cancer receiving OATs that is composed of different educational sections aimed at assessing general patient knowledge about their treatment protocol and drug information (eg, side effects), skills in the management of therapy, possible strategies to manage nonadherence occurrences, and a specific questionnaire section to evaluate patient comprehension [26]. Moreover, Omaki and colleagues [27] have developed a patient decision aid named “My Healthy Choices” to foster adherence to pain treatments, assessing the environmental and personal risks and setting patient treatment priorities.

Nevertheless, no study has been conducted on patients with MBC to foster medication adherence to OATs through the clinical care pathway based on designing and developing a decision support system (DSS), integrating risk predictive models and educational and training tools. The information embedded in a DSS solution designed and developed according to the needs of patients with MBC might enable users to be better informed, develop more accurate expectations of the benefits and harms, and increase participation in the decision-making processes and medication adherence [28]. Evidence shows that, when implemented on web or mobile apps, DSS may support patients and physicians by improving adherence to medical treatments [27,29,30].

### The Pfizer Project (65080791)

Drawing from the theoretical framework described above, we present and explain the study protocol of a prospective, randomized controlled study that is nested in a large-scale international project named “Enhancing Therapy Adherence Among Metastatic Breast Cancer Patients” (Pfizer 65080791) aimed to develop a predictive model of nonadherence and an associated DSS and guidelines to foster patients’ engagement and therapy adherence among patients with MBC concerning oral chemotherapy, endocrine therapy, supportive care, and the treatment of comorbidities. Consistently, the Pfizer Project (65080791) has been organized into 2 different studies to achieve the key goal. A retrospective study has been designed and a model has been developed to predict adherence to OATs, which use existing physiological, clinical, and QoL data available in the European Institute of Oncology (IEO; Milan, Italy). More in-detail, multimodal retrospective data have been retrieved from patient electronic health records by using natural language processing in a sample of 2750 patients with MBC (from 2010 to 2020). Data included in the analysis have been sociodemographic variables, diagnosis, biochemical and medical tests, procedures and medical history, treatment programs, treatment side effects, comorbidities, and familiarities. Concerning adherence to the treatment protocols, data included the following dimensions: initiation of the treatment, interruption of treatment, and skipped treatment doses. Furthermore, a prospective study is designed to assess the effectiveness of a DSS web-based solution and to enrich the predictive power of the machine learning model to forecast adherence behavior in patients with MBC. The tuning of the model permits adding additional predictors (personality traits, self-efficacy for coping with cancer, sense of coherence, pain, anxiety, depression, risk perception, and QoL) known to influence medication adherence behavior and that are not available retrospectively [10]. These data are used to improve the predictive power of the machine learning model and its capacity to profile patients’ adherence behaviors and to provide an individual risk value of nonadherence.

## Methods

### Primary End Point Analysis

The primary end point was to assess the effectiveness of the DSS web-based solution and the machine learning web application in promoting adherence to OATs in a sample of 100 patients with MBC at 3 months. The adherence is evaluated using the number of pills taken divided by the number of pills prescribed. Further, the adherence is assessed using behavioral measures: the 8-item Morisky Medication Adherence Scale (MMAS-8) [31] and Adherence Attitude Inventory (AAI) [32].

### Secondary End Point Analysis

The secondary end points were to identify clinical (comorbidities, presence of pain, tumor type, type of treatment), psychological (personality traits, anxiety, depression, self-efficacy for coping with cancer, sense of coherence, and risk perception), and QoL variables predicting patients’ adherence to OATs. These variables are used as predictors for evaluating nonadherence to OATs among patients with MBC and for enriching the preliminary version of a machine learning model developed in the retrospective study. Our initial machine learning models that are evaluated as an intervention in this study were built based on variables extracted from clinical notes by using natural language processing. Once the data collection for this study is complete, we will also use the data collected to improve our initial machine learning models. Data for the secondary end points are collected using the European Organization for Research and Treatment of Cancer QoL Questionnaire Core 30 (EORTC-QLQ-C30) [33,34], European Organization for Research and Treatment of Cancer QoL 23-item Breast Cancer-specific Questionnaire (EORTC-QLQ-BR23) [35], and the Brief Pain Inventory (BPI) [36]. To evaluate psychological variables, we used the State-Trait Anxiety Inventory (STAI-Y) [37,38], Beck Depression Inventory-II (BDI-II) [39,40], Big Five Inventory (BFI) [41], Brief Italian version of Cancer Behavior Inventory (CBI-B/I) [42,43], 13-item Sense of Coherence scale (SOC-13) [44], and risk perception (using 2 visual analogue scales) [45,46].

### Study Design

#### Treatment Adherence DSS

The first version of the web-based DSS, namely, TREAT (treatment adherence support) was designed and developed in the first year of the Pfizer Project (65080791) by using a patient-driven approach. The development phase was systematized in 5 steps. First, a systematic review of the literature was conducted to explore the main issues related to the interventions fostering adherence in patients with breast cancer [47]. Second, 4 focus group studies with 19 patients with MBC (mean age [years] 55.95, SD 6.87; range 46-70) with different metastasis localizations were implemented in order to explore patients’ unmet needs related to the MBC disease and knowledge about the adherence, barriers, roadblocks, and resources related to OATs and to examine the role of technologies and decision support as aids fostering adherence behaviors during the care pathway [45]. The first and second steps informed the third step in which a preliminary set of mock-ups of TREAT using Wikimedia technology was shaped. TREAT was developed as a web-based application accessible by patients through a web link, without personal registration, and in Italian according to the patient’s user approach. The information contained in TREAT was constructed using the international guidelines for patients with MBC (eg, European School of Oncology-European Society for Medical Oncology 2nd international consensus guidelines for advanced breast cancer) [1,46]. Further, the information was organized using different setups (written text, figures, flowcharts, graphs, and tables). In the fourth step, this preliminary version was revised by an internal and multidisciplinary review group (oncologists, psychologists, technicians, and patients). Finally, the TREAT’s revised version was translated and published.

**Figure 1:**
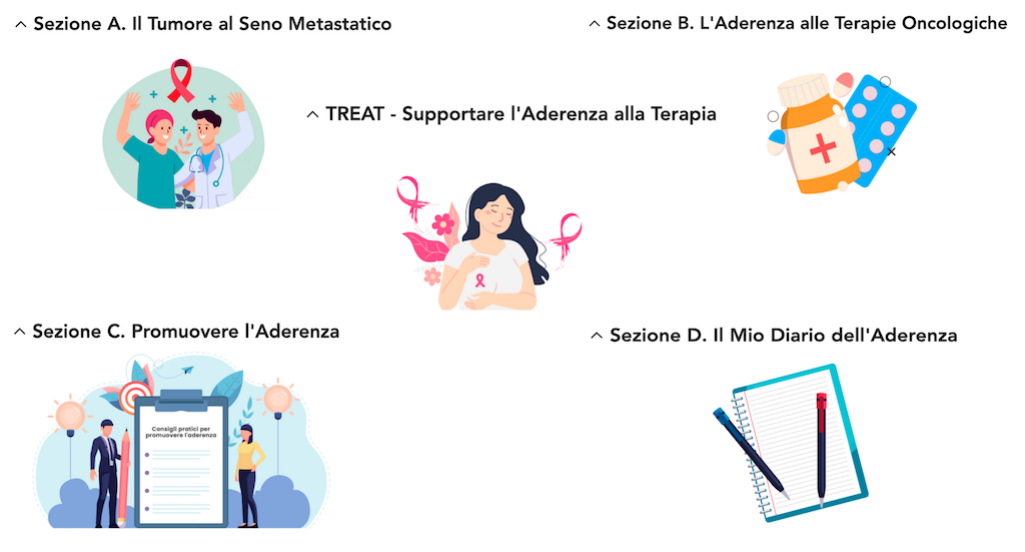
The 4 main sections of the decision support system in Italian. Section A: metastatic breast cancer; Section B: adherence to cancer therapies; Section C: promoting adherence; Section D: my adherence diary. The screenshot with the decision support system, namely, “TREAT - Supporting Therapy Adherence” is shown in the center.

**Figure 2:**
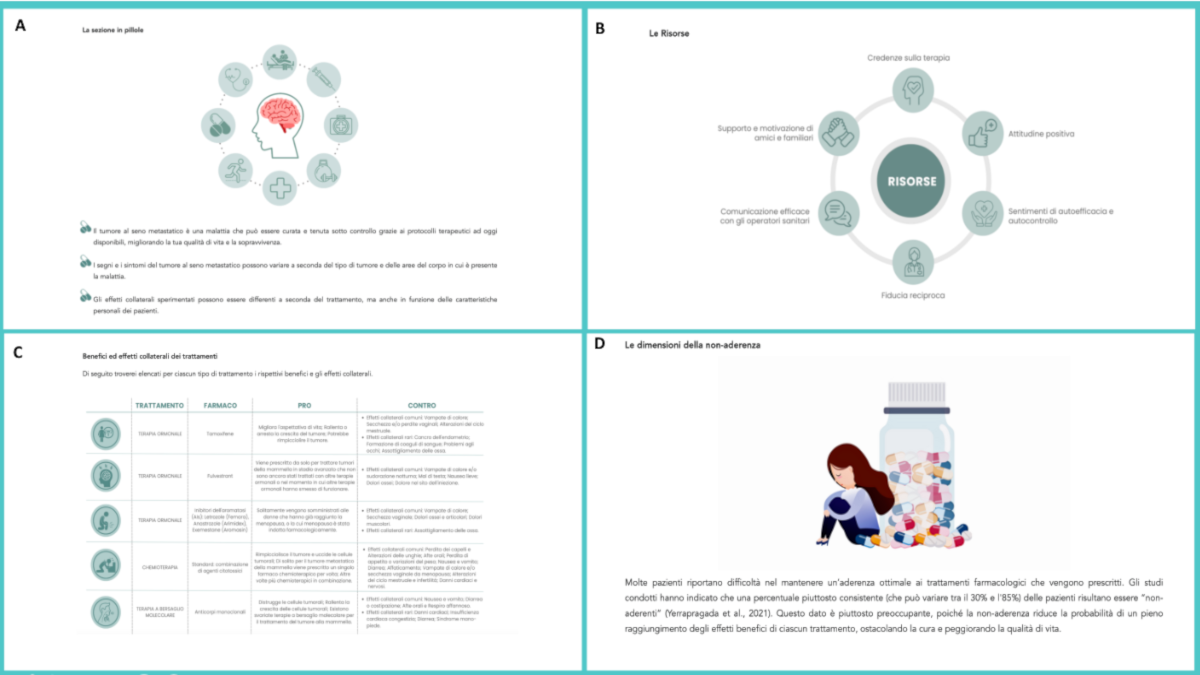
Screenshots of the decision support system, exemplifying some examples of the information nested in different sections of the system (A, B, C, D). A. “The Section on Pills” refers to a short summary enclosed at the end of each section of the decision support system reporting the key themes (renamed pills) discussed in the section. B. “Resources” is a flowchart on the main resources that can improve medication adherence. C. “Benefits and side effects of the treatments” is a table that shows the action of each anticancer treatment, type of drug, expected benefits, and side effects. D. “The adherence dimension” is a written text that informs about the dimension of nonadherence in the population with cancer.

Currently, TREAT is organized into 4 key sections. Section A on metastatic breast cancer provides information on MBC disease (eg, definition, clinical management), physical consequences (eg, pain, weight loss, lack of energy), psychological consequences (eg, anxiety, depression, fatigue), anticancer treatments and the associated side effects, and benefits that can be experienced during the care pathway. Section B on adherence to cancer therapies discusses the meaning of nonadherence and its consequences, incidence in the population with cancer, and determinants of medication adherence according to the World Health Organization’s approach. Section C on promoting adherence provides information about the resources (eg, personal beliefs, social support, trust in the health care providers), barriers (eg, distress, inadequate knowledge) of medication adherence, and the different available interventions (eg, educational, affective, behavioral) to promote patients’ adherence. Further, TREAT delivers specific self-managed suggestions to mitigate potential risk factors associated with nonadherence and an example of a specific training based on a goal-setting approach. In Section D, that is, My Adherence Diary, patients are invited to write a free-text diary reporting doubts, concerns, thoughts, and behaviors related to their disease and therapies, sharing this information with their oncologists in the following clinical consultation (Figures 1-2).

We also developed a machine learning application, which will be tested with the DSS. This application focuses on models to predict adherence risk factors and used data extracted from the electronic health records of the IEO. Specifically, the machine learning models focused on 2 outcomes: short-term and long-term side effects as well as physical status and comorbid conditions. We evaluated the models predicting performance by utilizing metrics such as area under the curve, precision, recall, sensitivity, specificity, κ, and positive and negative predictive values. Based on these criteria, we identified the top performing models, which were integrated into a web application built using the Shiny open source framework for the R statistical language (R Core Team) [48]. We designed this application for shared decision-making sessions between patients and oncologists. It also uses the Shapley interpretable machine learning algorithm, which offers insights into the specific risk factors that played a role in predicting outcomes for individual patients.

#### Participants

A sample of 100 patients enrolled consecutively in May 2023 in the IEO at the Division of Medical Senology with an MBC diagnosis. The enrollment started in May. Specifically, the observed outcomes of 50 patients with MBC exposed to the DSS (experimental group) and 50 patients with MBC not exposed to the intervention (control group) are evaluated.

#### Inclusion and Exclusion Criteria

The inclusion criteria were female patients with MBC, 18 years of age and older, with a prescription of oral treatment (eg, oral chemotherapy, endocrine therapy, cyclin-dependent kinase 4/6 inhibitors), having internet access and a personal smartphone or tablet, and ability to read and sign informed consent. The exclusion criteria were the presence of any primary psychiatric or neurological conditions.

### Procedure

#### Randomization

Patients who signed the informed consent are given a unique identifier and assigned to either the control or intervention arm in a 1:1 ratio. First, the system asks to confirm all the inclusion and exclusion criteria. Then, an independent researcher generates a random sequence using the statistical language R (R Core Team 2020). Our randomization schedule involves an undisclosed blocking size that the data science team calculates without stratification. A patient is considered randomized when the randomization system assigns the patient an identification number according to a pre-established randomization list. We have created 2 different groups: the experimental group (n=50), in which each participant receives the link for the DSS and is trained to use the DSS for 3 months; and the control group (n=50), in which each patient receives standard care and suggestions bolstering adherence. Among all participants, the personal nonadherence risk is calculated through the preliminary version of the machine learning model to predict patients’ nonadherence behavior. 

#### Recruitment and Follow-Up

Patients admitted to the Division of Medical Senology of the IEO are enrolled by the oncologist during the formal clinical consultation. Patients are enrolled even if they already had oral treatments in the past and they are switching to a new one. The patients who show interest in this research have a phone call with a trained psychologist to receive further information about the Pfizer study. If patients decide to participate in this study, the psychologist plans a consultation to receive informed consent and a preliminary assessment. Patients are informed that TREAT will not replace the clinical consultation. However, it should help manage oral treatment and improve adherence through education using evidence-based information. Patients may decide to discontinue their participation in the trial without any penalties. Patients who refuse to participate in this study are asked to complete a short refusal survey regarding the main reasons for not participating and their demographic information in order to assess the potential differences among the participants. Furthermore, patients have to contact the pertaining division for any necessity. Data are collected by the REDCap (research electronic data capture) platform and stored centrally by the IEO.

### Measures

#### Assessment of Nonadherence to OATs

This study uses the operational definition of adherence provided by the International Society for Pharmacoeconomics and Outcomes Research Medication Compliance and Persistence Work Group, which defines adherence as “the extent to which a patient acts in accordance with the prescribed interval and dose of a dosing regimen” [49]. Coherently, the nonadherence to OATs is evaluated using a prospective method [15] by weekly medication diaries and 2 self-reported measures.

#### Adherence Medication Diary

A weekly paper diary is given to patients to monitor their medication intake. The diary will assess if medications are taken as prescribed and the possible reasons for not taking pills (eg, side effects, forgetfulness, no pill refill). The data collected refer to the number of pills not taken per week and the number of pills established according to the patient medical prescription protocol per week as well as planned interruptions. Further, the diary evaluates and monitors the side effects, their intensity (from 0 to 10) associated with the therapy intake, and their emotions, thoughts, behaviors, and consequences.

#### MMAS-8 Self-Report Questionnaire (© 2006 Donald E Morisky)

The MMAS-8 scale [31,50,51] is an 8-item self-report questionnaire evaluating treatment adherence (forgetfulness, medication-taking behavior, adverse effects, and problems) (Cronbach α=.83). The first 7 items have dichotomous responses (0=Yes, 1=No), and the last includes a 5-point Likert scale response [52].

#### AAI Self-Report Questionnaire

The AAI is a 28-item self-report questionnaire on a 5-point Likert scale response (from “does not fit” to “perfect fit”) evaluating treatment adherence (Cronbach α=.80) [32]. The AAI is organized into 4 subscales: cognitive functioning (evaluating the ability to remember issues and tasks related to adherence in the short and long term), patient-provider communication (evaluating thoughts, attitudes, feelings, and ideas related to adherence between patient and medical provider), self-efficacy (evaluating the belief in one’s ability to adhere to medication and a history of similar success), and commitment to adherence (evaluating the determination to overcome obstacles to achieve adherence).

#### Demographic, Clinical, Emotional, and QoL Assessments

Considering that adherence to OATs is explained by a set of mutual and interconnected determinants, a comprehensive pool of self-reported measures is administered to identify the psychological predictors of nonadherence in patients with MBC.

#### Patient Demographic and Clinical Variables

Age, gender, education, marital status, cancer diagnosis and staging, oncological treatments, BMI, alcohol and smoking habits, and comorbid medical disorders are collected through electronic medical records.

#### STAI-Y Self-Report Questionnaire

The STAI-Y is a 40-item self-report questionnaire on a 4-point Likert scale (Cronbach α=.89). The STAI-Y evaluates both trait anxiety (20 items) and state anxiety (20 items) [37,38].

#### BDI-II Self-Report Questionnaire

The BDI-II is a 21-item self-report questionnaire on a 4-point Likert scale response evaluating depression (Cronbach α=.89). The BDI can be administered to adults and adolescents aged 13 years and older [39,40].

#### CBI-B/I Self-Report Questionnaire

The CBI-B/I is a 12-item self-report questionnaire on a 7-point Likert scale response (from not all confident to totally confident) evaluating self-efficacy for coping in patients with cancer (Cronbach α=.84). The CBI-B/I is composed of 4 subscales: coping and stress management, maintaining independence, managing affect, and participating in medical care [42,43].

#### SOC-13 Self-Report Questionnaire

The SOC-13 is a 13-item self-report questionnaire on a 7-point Likert scale response (Cronbach α=.76). The SOC-13 is composed of 3 subscales: comprehensibility, manageability, and meaningfulness [44].

#### BPI Self-Report Questionnaire

The BPI is a 9-item self-report questionnaire evaluating pain intensity during the past 24 hours (Cronbach α=.91) [36].

#### EORTC-QLQ-C30 Self-Report Questionnaire

The EORTC-QLQ-C30 is a self-report questionnaire composed of 28 items on a 4-point Likert-type scale (ranging from not at all to very much) and 2 items, that is, general global health status and QoL on a 7-point Likert-type scale (ranging from very poor to excellent) [33,34]. The EORTC-QLQ-C30 provides information on 3 areas: functional (physical, role, cognitive, emotional, and social), symptoms (appetite loss, fatigue, pain, nausea, constipation-diarrhea, dyspnea, and insomnia), and global health status or QoL (Cronbach α=.85) [32,33]. Further, the EORTC-QLQ-B R23 for patients with breast cancer has been used previously (Cronbach α=.87) [34,35].

#### BFI Self-Report Questionnaire

The BFI is a 44-item self-report questionnaire on a 5-point scale (from disagree strongly to strongly agree) that assesses 5 dimensions of personality: openness to experience (Cronbach α=.78), conscientiousness (Cronbach α=.81), extraversion (Cronbach α=.87), agreeableness (Cronbach α=.81), and neuroticism (Cronbach α=.82) [41].

#### Risk Perception

Risk perception is evaluated using 2 visual analogue scales (from 0 to 100): one for the objective and one for the subjective risk perception. These items were developed using the Weinstein approach [45,46].

### Timeline

There are 3 assessment time points. At the baseline (T0), all patients fill a set of validated measures (MMAS-8, AAI, STAI-Y forms I and II, BDI-II, CBI-B/I, SOC-13, BPI, EORTC-QLQ-C30, EORTC-QLQ-BR23, BFI, and visual analogue scale). The expected time to complete all the given questionnaires at baseline is approximately 40 minutes. Access to DSS is given to the experimental group for 3 months. At T1 and T2, the following questionnaires are filled: MMAS-8, AAI, STAI-Y form I, BDI-II, CBI-B/I, EORTC-QLQ-C30, and EORTC-QLQ-BR23. Further, all patients have to fill a weekly adherence medication diary for 3 months. Variables that are not sensitive to change, for example, personality (BFI) and anxiety traits (STAI-Y form II) are collected only at T0. Each month, all participants receive a brief telephone interview in which they are monitored for adherence to the research protocol. Two psychologists perform the monthly telephone interview to monitor the filling out of the questionnaires and the medication adherence diary supporting patients with MBC in this task, barriers, and concerns related to the study participation. At T3 (3 months), all behavioral and psychological measures are filled, and an interview (online or vis-à-vis) is performed.

### Calculation of Sample Size

The sample size calculation was based on estimates for the effectiveness of TREAT as the primary outcome, assuming that the final analysis would be performed with a 2-sample 2-sided *t* test, a power of 96.4%, an α of .05, a standard deviation of 1.2, and a minimal difference in outcomes of 0.51 (effect size of 0.42) based on the patients’ self-reported satisfaction with the treatment decision [53]. Under these considerations, the final sample size was calculated to be 100 patients with MBC, with 50 individuals per group (50 patients with MBC exposed to DSS and 50 patients with MBC not exposed to the intervention). This sample size is expanded to 120 when a 20% attrition rate is used.

### Statistical Considerations

#### Exploratory Analysis

Our exploratory analysis will start with a visual exploration of all variables to evaluate the frequency, percentage, and near-zero variance for categorical variables (eg, gender, cancer stage); distribution for numeric variables (eg, age); and their corresponding missing value patterns [54]. Near-zero variance is found when a categorical variable has a small percentage of a given category and will be addressed by recategorization. We will consider variable transformations such as logarithmic, Box-Cox, or categorization of numeric variables that do not present a normal distribution. Missing values will be handled through imputation algorithms followed by sensitivity analyses to verify whether our results are stable with and without imputation [53]. Comparisons for the exploratory analysis will be conducted through analysis of variance (2-sided *t* tests being a category of analysis of variance) and chi-square tests (Fisher exact test when any cell presented a frequency below 5).

#### Propensity Score

For residual confounding after randomization, we will use propensity scores based on inverse probability of treatment weighting [55]. We will first assess the covariate balance between control and intervention groups through standardized mean differences and differences in proportion. Covariates will include the patient demographic and clinical variables. Values greater than 0.1 will signal an imbalance in covariates. Next, we will use inverse probability of treatment weighting generated through logistic regression to balance the covariates [56]. Once we achieve covariate balance, we will estimate the effect for each outcome by using a double robust approach.

#### Generalized Linear Models

Additional analyses will be run using generalized linear models with a Gaussian distribution family (multiple linear regression) being adjusted for randomization baseline imbalances. These models will evaluate the association between all previously reported outcomes (the number of pills taken during the prescribed interval, MMAS-8, AAI, STAI-Y, BDI-II, CBI-B/I, SOC-13, BPI, EORTC-QLQ-C30, EORTC-QLQ-BR23, BFI, and risk perception) and the intervention (TREAT and machine learning web application), accounting for the baseline differences. We will build one model for each combination of predictor and outcome, where the outcome will be the dependent variable (Y) and the predictor will be the independent variable (X), adjusted for the covariates unbalanced at baseline. We will also evaluate models with a Poisson or negative binomial distribution for non–normally distributed outcomes. Results will be reported as predicted means along with 95% CIs.

### Ethics Approval

This study is compliant with the recommendations outlined in the Helsinki Declaration [57] and the Council for International Organizations of Medical Sciences guidelines [58], as well as with the principles of biomedical ethics reported in the Belmont report [59]. This study presents a fair balance between risks and benefits for study participants and future patients affected by the same condition. No physical risks directly related to participation in the research are expected. Although psychological risks are not expected, in case these raised from participation, psychologists responsible for the study will promptly intervene and take care of the patient. Regarding the benefits, given the impact of nonadherence on clinical outcomes, finding effective ways to increase treatment adherence is particularly relevant for the community of patients with MBC, thus playing a role in the improvement of the patient’s general well-being. The principle of self-determination is also respected. A devoted informed consent form is signed by participants before participation. Signing the informed consent form is preceded by a dialogic consent process necessary to ensure informed, voluntary, and awareness of participation in research. Regarding the respect for privacy, this study is performed according to the General Data Protection Regulation (Regulation EU, 2016/679) [60]. All data are collected in a pseudoanonymized form. Data are treated confidentially and used only by the collaborators in this study for scientific purposes related to what is stated in the research protocol. The retrospective study was approved by the IEO ethics committee (R1595/21-IEO 1704). The prospective study was approved by the IEO ethics committee (R1786/22-IEO 1907).

## Results

The recruitment process started in May 2023 and is expected to be concluded on December 2023. Data analysis will be performed between 2023 and 2024. This project has been funded by a Pfizer grant: Enhancing therapy adherence among patients with metastatic breast cancer (65080791).

## Discussion

### Expected Findings

Adherence to anticancer treatment is fundamental in the clinical management of patients with MBC, as adherence can lead to a series of crucial clinical outcomes (eg, prolonged survival time, better monitoring and management of side effects, improvement of QoL) [1-6]. Studies on implementing machine learning to foster medication adherence as well as the development of shared DSSs are quite recent [23].

We believe that our study might achieve 3 milestones in the clinical care of patients with MBC with regard to adherence to OATs. Concerning the primary end point, implementing a shared and integrated DSS web-based solution might be an essential strategy for patients with MBC to enhance their health knowledge and understanding of medication nonadherence issues and to learn behavioral strategies to overcome individual, disease-related, and organizational barriers impacting adherence behaviors. This expected result is coherent with the results of other studies on patient decision tools, highlighting that such engines might improve patients’ participation and health knowledge in the care pathway [61]. Regarding the secondary end point, this prospective study will contribute to refining the initial risk-predictive model of adherence attained from the retrospective study, informing about specific (inner and external) predictors of adherence behavior during the MBC care pathway that were not considered in the preliminary version of the model. This should provide a more comprehensive and systematic definition of medication adherence for OATs in patients with MBC, defining an interrelated and evidence-based list of predictors. Data will define a final risk-predictive model of adherence and enable the identification of specific patient populations at risk of nonadherence. The earlier identification of patients with poor adherence using our machine learning web application will permit the development of tailored psychological and behavioral interventions to foster adherence by dealing with individual barriers and needs according to the risk profile. Finally, the information retrieved by the risk-predictive models might support oncologists’ treatment decisions, allowing them to better understand the hindered reasons behind a complex adherence trajectory.

### Limitations of This Trial

Despite the key contributions of this study in the field of the medication adherence to OATs among patients with MBC, some limitations have to be acknowledged. First, the questionnaires used to refine our risk-predictive model might cause cognitive burden and general fatigue among participants. However, a monthly telephone interview has been introduced as a mitigation risk strategy. The interview is aimed to support participants in filling out the questionnaires and medication adherence diary and to manage barriers and concerns related to this study. Related to this aspect, the second limitation concerns the recall bias that might be generated using self-report measures. Further, self-report measures might overvalue adherence compared to the other prospective methods such as the Medication Event Monitoring System. However, the weekly medication diary method, in which patients have to report a detailed and systematic description of pill counts, side effects, and barriers related to the management of the therapy and missed doses, can aid in providing a more comprehensive picture of medication adherence. Moreover, as highlighted by Shah and colleagues [15], the diary has a higher accuracy and minor recall bias and permits the evaluation of subjective information (eg, emotions, thoughts, worries, behaviors, expectations) related to the medication coherently with the processual model of the adherence used in this research protocol [8]. Third, a monthly follow-up and a 3-month end point have been established, considering the type of diagnosis, high variation in the prognosis, and shifting in the lines of treatment due to cancer progression or declining physical functioning. This short end point might affect a deep understanding of the efficacy of our DSS in the long term. However, previous studies have used correspondent follow-ups and end points [13]. Fourth, as observed by other studies on patients with MBC [14], an additional risk that should be considered is related to the overestimated adherence rate. Patients participating in clinical trials are highly motivated, possibly leading to greater adherence to the OATs prescribed. Fifth, the initial version of the machine learning web application was not able to directly predict patient adherence. Instead, it predicted adherence risk factors, including short-term and long-term side effects, as well as physical status and comorbid conditions. This is a limitation of the data we used to build this initial version of the model, as it was not possible to reliably identify adherence patterns. However, predicting adherence risk factors can still provide insights that can assist in improving OAT adherence during shared decision-making sessions between physicians and patients.

### Conclusions

Considering the poor evidence on OATs and the need to develop validated instruments to evaluate medication adherence to improve patient clinical outcomes [14,16], the development of integrated and shared DSSs able to foster adherence behaviors and to profile patient adherence risks might have key impacts on clinical practice and patient health-related QoL, thereby enabling the early identification of high-risk populations and enriching knowledge about the implementation of machine learning models in clinical practice.

## Abbreviations

AAI: Adherence Attitude Inventory
BDI-II: Beck Depression Inventory-II
BFI: Big Five Inventory
BPI: Brief Pain Inventory
CBI-B/I: Brief Italian version of Cancer Behavior Inventory
DSS: decision support system
EORTC-QLQ-BR23: European Organization for Research and Treatment of Cancer Quality of Life 23-item Breast Cancer-specific Questionnaire
EORTC-QLQ-C30: European Organization for Research and Treatment of Cancer Quality of Life Questionnaire Core 30
IEO: European Institute of Oncology
MBC: metastatic breast cancer
MMAS-8: 8-item Morisky Medication Adherence Scale
OAT: oral anticancer treatment
QoL: quality of life
REDCap: research electronic data capture
SOC-13: 13-item Sense of Coherence scale
STAI-Y: State-Trait Anxiety Inventory
TREAT: treatment adherence support

## Data Availability

Data will be available from the corresponding author on reasonable request.
