# Correction: A Machine Learning Model to Predict Patients’ Adherence Behavior and a Decision Support System for Patients With Metastatic Breast Cancer: Protocol for a Randomized Controlled Trial

**DOI:** 10.2196/55928

**Published:** 2024-01-15

**Authors:** Marianna Masiero, Gea Elena Spada, Virginia Sanchini, Elisabetta Munzone, Ricardo Pietrobon, Lucas Teixeira, Mirtha Valencia, Aline Machiavelli, Elisa Fragale, Massimo Pezzolato, Gabriella Pravettoni

**Affiliations:** 1 Department of Oncology and Hemato-oncology University of Milan Milan Italy; 2 Applied Research Division for Cognitive and Psychological Science European Institute of Oncology IRCCS Milan Italy; 3 Division of Medical Senology European Institute of Oncology IRCCS Milan Italy; 4 SporeData Inc Durham, NC United States

In “A Machine Learning Model to Predict Patients’ Adherence Behavior and a Decision Support System for Patients With Metastatic Breast Cancer: Protocol for a Randomized Controlled Trial” (JMIR Res Protoc 12(1); e48852) the authors noted one error.

In the Acknowledgments, the last sentences appeared as follows:

The 8-item Morisky Medication Adherence Scale content, name, and trademarks are protected by US copyright and trademark laws. Permission for use of the scale and its coding is required. A license agreement is available from MMAS, LLC. The property/identifier issue/registration date/categories are as follows: US registration 5,837,273 August 20, 2019, Trade/Service Mark; US registration TX-8-285-390 June 12, 2016, Copyright; and US registration TX-8-632-533 September 21, 2018, copyright.

This project has been funded by a Pfizer grant: Enhancing therapy adherence among patients with metastatic breast cancer (65080791).

This has been changed to appear as follows:

The MMAS-8 Scale, content, name, and trademarks are protected by US copyright and trademark laws. Permission for use of the scale and its coding is required. A license agreement is available from MMAR, LLC., www.moriskyscale.com.

This project has been funded by a Pfizer grant: Enhancing therapy adherence among patients with metastatic breast cancer (65080791).

The correction will appear in the online version of the paper on the JMIR Publications website on January 15, 2024 together with the publication of this correction notice. Because this was made after submission to PubMed, PubMed Central, and other full-text repositories, the corrected article has also been resubmitted to those repositories.

